# Praying and Dieting

**Published:** 1886-05

**Authors:** 


### PRAYING AND DIETING.

Rev. J. T. Clymer in his admirable little work on "Food and Morals" sees clearly the means to be tried in the following case and a great many like it. In fact it points to the remedy in many evils: "A father, by prayer and precept and flogging, had done his best to reform his boy, whose staple diet was meat and sausage and pie and cake at his meals, with lunch between. The family physician said to the father, 'If you will put a leach back of each of your boy's ears once a week for a month you will do more to reform him than your preaching and pounding will do in a year.' The father asked for the philosophy of this prescription. 'Why,' said the doctor, 'your boy has bad blood and too much of it; he *must* behave badly or he would burst.' 'Then,' said the father, 'I'll change his diet from beef and pie to hominy and milk.' In three months thereafter a better boy of his age could not be found in the neighborhood. The acrid, biting, evil blood had not become food for leeches, but had done its wicked work and passed away, and a cool order, blander power, safer blood had been supplied from sweeter, gentler food services."

